# Interleukin‐7 aggravates myocardial ischaemia/reperfusion injury by regulating macrophage infiltration and polarization

**DOI:** 10.1111/jcmm.16335

**Published:** 2021-09-28

**Authors:** Mengwen Yan, Yaliu Yang, Ying Zhou, Changan Yu, Rui Li, Wei Gong, Jingang Zheng

**Affiliations:** ^1^ Department of Cardiology China‐Japan Friendship Hospital Beijing China; ^2^ Central Laboratory of Cardiovascular Disease China‐Japan Friendship Hospital Beijing China; ^3^ Department of Health Care China‐Japan Freindship Hospital Ministry of Health Beijing China; ^4^ Emergency and Critical Care Center Beijing Anzhen Hospital Capital Medical University Beijing China; ^5^ Beijing Institute of Heart, Lung, and Blood Vessel Diseases Beijing China; ^6^ Department of Cardiology China‐Japan Friendship School of Clinical Medicine Peking Union Medical College Chinese Academy of Medical Sciences Beijing China; ^7^ Department of Cardiology Peking University China‐Japan Friendship School of Clinical Medicine Beijing China

**Keywords:** interleukin‐7, ischaemia/reperfusion injury, macrophage, myocardial

## Abstract

Interleukin (IL)‐7 is known to enhance the macrophages cytotoxic activity and that macrophages play a pivotal role in the development and progression of myocardial ischaemia/reperfusion (I/R) injury. However, the effects of IL‐7 on macrophages infiltration and polarization in myocardial I/R injury are currently unclear. This study aimed to evaluate the effects of the IL‐7 expression on myocardial I/R injury and their relationship with macrophages. The data showed that IL‐7 expression in mouse heart tissue increases following I/R injury and that IL‐7 knockout or anti‐IL‐7 antibody treatment significantly improve I/R injury, including reduction in myocardial infarction area, a serum troponin T level decreases and an improvement in cardiac function. On the other hand, recombinant IL‐7 (rIL‐7) supplementation induces opposite effects and the anti‐IL‐7 antibody significantly reduces the cardiomyocyte apoptosis and macrophage infiltration. rIL‐7 cannot directly cause apoptosis, but it can induce cardiomyocyte apoptosis through macrophages, in addition to increase the macrophages migration in vitro. Anti‐IL‐7 antibody affects the cytokine production in T helper (Th) 1 and Th2 cells and also promotes the macrophages differentiation to M2 macrophages. However, anti‐IL‐7 antibody does not reduce the M1 macrophage number, and it only increases the ratio of M2/M1 macrophages in mice heart tissues after I/R injury. Taking together, these data reveal that IL‐7 plays an intensifying role in myocardial I/R injury by promoting cardiomyocyte apoptosis through the regulation of macrophage infiltration and polarization.

## 1 INTRODUCTION

Coronary artery recanalization, including coronary artery bypass grafting, percutaneous coronary intervention and thrombolytic therapy, is currently the main treatment for myocardial infarction. Coronary artery recanalization is able to clear the narrow or even occluded coronary lumen, but ischaemia‐reperfusion (I/R) injury has been the most important obstacle to the blood reflow treatment.^1^ Inflammation caused by the infiltration of immune cells, such as macrophages, plays a pivotal role in the development and progression of myocardial I/R injury.^2^, ^3^

Interleukin (IL)‐7 is produced mainly by thymic stromal cells and can be secreted by other cells, such as foetal liver cells, bone marrow stromal cells, spleen cells, macrophages and dendritic cells.^4^ Under pathological conditions, some tumours and endothelial cells can also produce IL‐7.^5^ Previous research on IL‐7 mainly focused on its effects on T and B cells. IL‐7 has been found to be an essential cytokine for growth, survival, differentiation and appreciation of B cells.^6^ In addition, these studies found that IL‐7 played a key role in the development, proliferation and homeostasis of T cells.^7^ However, the other IL‐7 functions were also been evaluated by the researchers. Among them, we have paid attention to the fact that IL‐7 enhances the macrophage cytotoxic activity,^8^, ^9^ induces monocyte macrophages to secrete a variety of pro‐inflammatory cytokine factors (MCP‐1, MIP, IL‐1β, among others),^10^ regulates the interaction between various components of the inflammatory process ^11^ and increases the expression of monocyte‐derived inflammatory chemokine receptors (CCR), such as CCR1, CCR2 and CCR5.^12^

Monocytes/macrophages are the inflammatory cells with the longest residence time in repairing the myocardial tissue damage after I/R injury.^2^, ^3^ In the early stage of inflammation, these cells play a role primarily in the pro‐inflammatory response and swallowing and promote the regeneration and remodelling of granulation tissue in the inflammation middle and late stages.^12^, ^13^ Importantly, in myocardial I/R injury, inhibiting excessive inflammation can reduce myocardial necrosis, protects vascular endothelial function and reduces infarct area.^14^ Besides, it helps to reduce myocardial I/R injury by regulating chemotaxis and macrophages polarization.^15^, ^16^ However, it is unknown whether IL‐7 can affect myocardial I/R injury by affecting macrophages invasion and polarization.

In this study, we evaluated the IL‐7 expression in mice heart tissues following myocardial I/R injury. In addition, we investigated the IL‐7 effects on myocardial I/R injury and on chemotaxis and polarization of macrophage by neutralizing endogenous IL‐7 and supplementing exogenous IL‐7 in mice. The data showed that IL‐7 aggravated myocardial ischaemia/reperfusion (I/R) injury by regulating macrophages infiltration and polarization.

## 2 MATERIALS AND METHODS

### 2.1 Animals and Myocardial I/R injury in vivo

A total of 81 wild‐type (WT) C57BL/6 mice (8‐10 weeks) and 6 IL‐7 knockout (IL‐7‐/‐) C57BL/6 mice (8‐10 weeks) were used in the present study. WT C57BL/6 mice were purchased from Vital River Laboratory Animal Technology Co. Ltd (Beijing, China). IL‐7‐/‐ C57BL/6 mice were generated as previously described.^17^ Animal procedures were performed with the consent from the China‐Japan Friendship Hospital ethics committee. The I/R mice model was established as described previously.^18^ Briefly, mice were anaesthetized with ketamine (50 mg/kg) and pentobarbital sodium (50 mg/kg). After the mouse thoracic cavity was opened, the left coronary artery (LCA) was quickly found and the ligature was performed (mice in the sham group were not submitted do LCA ligature). The left ventricle was kept ischaemic for 30 minutes because of LCA ligature, which was subsequently removed for reperfusion. The mice were killed after reperfusion for 1, 4, 12, 24, 48 and 72 hours to obtain heart tissues.

### 2.2 Real‐time quantitative PCR

Total RNA in cells and tissues was extracted by an RNA extraction Kit (RC101‐01; Vazyme) and was reverse‐transcribed into cDNA using the PrimeScript RT reagent (RR047A, Taraka, Japan). Then, 20 μL of RT‐qPCR system was prepared as described in the qPCR master mix kit instructions (A6001, Progema, USA). The gene relative expression was calculated by 2^‐ΔΔ^ *^C^* ^t^ method, and β‐actin was used as a loading control. The primers used for qPCR analysis are shown in Table 1.

**Table 1:** Sequence of qPCR primers

Gene	Sequence (5′‐3′)
IL‐7	Forward: TCTGCGAGTACCCTGTCATC
	Reverse: CCACTTCTGTCTAAGGAGCCAAT
MCP‐1	Forward: TAAAAACCTGGATCGGAACCAAA
	Reverse: GCATTAGCTTCAGATTTACGGGT
CD68	Forward: TGTCTGATCTTGCTAGGACCG
	Reverse: AGGAGAGTAACGGCCTTTTTG
MIP‐2	Forward: CCAACCACCAGGCTACAGG
	Reverse: GCGTCACACTCAAGCTCTG
TNF‐α	Forward: CCTGTAGCCCACGTCGTAG
	Reverse: GGGAGTAGACAAGGTACAACCC
IFN‐γ	Forward: ATGAACGCTACACACTGCATC
	Reverse: CCATCCTTTTGCCAGTTCCTC
IL‐4	Forward: CCCCAGCTAGTTGTCATCCTG
	Reverse: CAAGTGATTTTTGTCGCATCCG
IL‐13	Forward: CAGCCTCCCCGATACCAAAAT
	Reverse: GCGAAACAGTTGCTTTGTGTAG
iNOS	Forward: GTTCTCAGCCCAACAATACAAGA
	Reverse: GTGGACGGGTCGATGTCAC
CCL2	Forward: GGCATCCCTCTACCCAAGAC
	Reverse: GGGCGTTAACTGCATCTGGA
Arg1	Forward: CTCCAAGCCAAAGTCCTTAGAG
	Reverse: GGAGCTGTCATTAGGGACATCA
CD206	Forward: GCTTCCGTCACCCTGTATGC
	Reverse:TCATCCGTGGTTCCATAGACC
β‐actin	Forward: AGCCCATCCTTCGAGTACAAA
	Reverse: TCTTGGTGCGATAACTGGTGG

### 2.3 Western blot analysis

Total protein was extracted from tissues and cells using a Tissue Protein Extraction Reagent (78510, Thermo Fisher) and RIPA lysis buffer (89900, Thermo Fisher), respectively. A BCA kit (23227, Thermo Fisher) was used to determine the protein concentration. Then, 40 μg total protein was separated by 10% SDS‐PAGE and transferred to PVDF membranes (LC2002, Thermo Fisher), which were first blocked with 5% skimmed‐milk powder and subsequently labelled with primary antibodies against IL‐7 (AF407; R&D), IL‐7R (ab95024; Abcam, UK), Bax (ab32503; Abcam) and Bcl2 (ab196495; Abcam). The proteins were visualized with ECL solution (WBKLS0100, Beijing Xinjingke Biotechnologies Co., Ltd), followed by densitometry analysis using Imag J 3.0 (IBM). β‐actin was loaded as control.

### 2.4 Enzyme‐linked immunosorbent assay (ELISA) and myeloperoxidase (MPO) activity assay

After 24 hours of reperfusion, venous blood from mice was collected through the posterior orbital venous plexus to determine the serum troponin T level using an ELISA kit for troponin T (Mito Sciences). Next, the mice heart tissues were harvested to detect IFN‐γ (ab252352; Abcam), TNF‐α (ab208348; Abcam), IL‐4 (ab221833; Abcam) and IL‐13 (ab219634; Abcam) protein levels using an ELISA kit. The myeloperoxidase (MPO) level was detected using an MPO activity assay kit (ab105136; Abcam).

### 2.5 Endogenous IL‐7 neutralization and exogenous rIL‐7 repletion

Thirty minutes of ischaemia and 24 hours of reperfusion were chosen as treatment time‐points. The mice were randomly divided into sham, IgG, anti‐IL‐7, vehicle and rIL‐7 groups (eight mice in each group). In the sham group, the mice only had their thorax open, were not submitted to LCA ligature and received no treatment. In the IgG group, the mice were injected IV with 100 μg rat IgG2A isotype control (MAB006; R&D). In the anti‐IL‐7 group, the mice were injected IV with 100 μg mouse IL‐7 monoclonal antibody (MAB4071, R&D). In the vehicle group, the mice were injected IV with 50 μL sterile saline. Finally, in the rIL‐7 group, the mice were injected IV with 1μg IL7 recombinant mouse protein diluted in 50 μL sterile saline. After 25 minutes of ischaemia, mice in the IgG, Anti‐IL‐7, Vehicle and rIL‐7 groups were injected IV with the corresponding reagent and continued to perform ischaemia for 5 minutes and reperfusion for 24 hours.

### 2.6 Cardiac function and haemodynamic analysis

An animal electrocardiogram (ECG) record analyser (LS20, B&E TEKSYSTEMS, China) to measure the mice ECG for later calculation of the ejection fraction and fraction shortening of left ventricular (LV), LV end‐diastolic pressure (LVEDP) and maximal (LV + dp/dt_max_) according to the standard formulas.^19^

### 2.7 Myocardial infarction area measurement

The killed mice hearts were harvest, and the myocardial infarction area was detected using TTC staining. The preparation of heart tissue sections and TTC staining were performed as described previously.^20^ Image J software (National Institutes of Healt) was used to the measure the area of myocardial infarction per section. The myocardial infarction volume was calculated by multiplying the myocardial infarction area in each slice by the section thickness (2 mm).

### 2.8 Apoptosis analysis

TUNEL staining was used to determine the apoptosis level in the heart tissues as described previously.^18^ Briefly, the mice hearts were fixed with paraformaldehyde, embedded in paraffin and sliced into tissue sections. After dewaxing and hydration, the tissue sections were incubated with proteinase K to permeate the cells. Then, the sections were incubated with TUNEL reaction solution for 1 hour at 37°C followed by colour development with DAB solution. All reagents and procedures for the TUNEL assay came from the TUNEL Cell Apoptosis Detection Kit (TA201‐02; TRANSGEN). Moreover, a Caspase 3 activity assay kit (C1115; Beyotime) was used to measure the Caspase 3 activity in vitro following the manufacturer's instructions. The cells were harvested, washed with cold PBS and fixed with cold 70% ethanol at 4°C for 1 hour. Then, the cells were resuspended using cold PBS after removing the ethanol by centrifugation. As stated by the manufacturer's instructions (E606336, Sangon Biotech), 15 minutes after adding the labelling reagent, the cells were collected and resuspended with PBS to be detected. Finally, the apoptosis level was also assessed by measuring Bax and Bcl2 expression by Western blot.

### 2.9 Mouse neonatal cardiomyocytes isolation

Neonatal mice (1 day old) were killed by cervical dislocation to collect the heart tissues as previously described.^21^ Next, the heart tissues were cut into small pieces and incubated with trypsin/EDTA solution (25200056; Thermo Fisher) for 30 minutes at 4°C. Then, the tissues were washed and the incubation was stopped by adding equal volume of DMEM medium (10567022; Gbico) containing 20% foetal bovine serum (FBS; 16140071, Gbico). After centrifugation, the tissues were collected and incubated with Liberase TH (5401151001; Roche) for 15 minutes at 37°C. The incubation with Liberase TH was repeated to ensure that all myocardial cells were harvest, which were subsequently filtered using a nylon cell strainer. Finally, neonatal cardiomyocytes were cultured in DMEM medium (10567022; Gbico) containing 10% FBS at 37°C with 5% CO_2_.

### 2.10 Treatment of mouse neonatal cardiomyocytes

After 72 hours of culturing neonatal mouse cardiomyocytes, 1 × 10^6^ of these cells were seeded in the lower chamber of the polycarbonate insert cell culture device (140640, Thermo Fisher, USA). Subsequently, different concentrations of rIL‐7 were added to the culture medium for 24 hours and then the cells were collected for apoptosis evaluation.

In a co‐culture system of macrophages and cardiomyocytes, 1 × 10^6^ macrophages were seeded in the upper chamber of polycarbonate insert cell culture device and 1 × 10^6^ neonatal cardiomyocytes were seeded in the lower chamber of that same device. Subsequently, different concentrations of rIL‐7 were added to the culture medium for 24 hours and then the cells were harvested for apoptosis analysis.

In a separate macrophage culture system, 1 × 10^6^ macrophages were seeded in the upper chamber of polycarbonate insert cell culture device. Next, the conditioned medium (CM) of macrophages was collected after stimulation with 50 ng/mL rIL‐7 for 24 hours. In a separate cardiomyocyte culture system, 1 × 10^6^ neonatal cardiomyocytes were seeded in the lower chamber of polycarbonate insert cell culture device and the conditioned medium (CM) described above was used to culture the cardiomyocytes for 24 hours. Finally, 24 hours after stimulation with or without 100 μmol/L H_2_O_2_, the cells were collected for apoptosis assessment.

### 2.11 Macrophages migration assay

Mouse primary peritoneal macrophages were prepared and cultured according to previously described methods.^22^ 1 × 10^6^ macrophages per well were seeded in the upper chamber of polycarbonate insert cell culture device (140644, Thermo Fisher, USA), and then, it was added nothing (control group), sterile saline (vehicle group), 50 ng/mL rIL‐7 (rIL‐7 group), 10 mg/mL rat IgG2a kappa Isotype Control (eBR2a)(14‐4321‐82, eBioscience) (IgG group) and 10 mg/mL CD127 monoclonal antibody (14‐1271‐82, eBioscience) (anti‐IL‐7R group) and incubated for 24 hours at 37°C. In the rIL‐7 and anti‐IL‐7R groups, 10 mg/mL CD127 monoclonal antibody was first added and incubated for 2 hours, and then, 50 ng/mL rIL‐7 was added and incubated for 24 hours. The cells were collected from the lower chamber, and the culture medium was removed. Then, they were washed three times with PBS, stained with 0.25% crystal violet for 25 minutes, slowly rinsed with sterile water, place on a sterile ultra‐clean bench to dry and finally the absorbance at 595 nm was measured.

### 2.12 Heart infiltrating cell isolation and flow cytometry analysis

Cardiac single cell suspensions were prepared as previously described.^23^ After removing red blood cells and counting cells, single cell suspensions were incubated for 30 minutes on ice with the following antibodies: anti‐CD45 (Pacific Blue, 50‐113‐811, Invitrogen, USA), anti‐CD11b (APC‐Cy7, MABF512MI, MilliporeSigma), anti‐Gr‐1 (APC, RM3005; Invitrogen). For intracellular staining, after the cells were fixed and permeabilized, they were incubated with the following antibodies for 30 minutes on ice: anti‐iNOS (PE, 14792; Cell Signaling Technology) and anti‐arginase 1 (FITC, 554001; BD). Flow cytometry was used to detect the fluorescence, and the data were analysed using the Flowjo software (v.7.6.2). Briefly, CD45 is a surface marker of white blood cells, Gr‐1 is a neutrophil surface marker and CD11b is a monocyte surface marker. Therefore, CD45 + Gr‐1‐cells were first selected and then CD11b+ cells were selected among these cells. After selecting CD45 + Gr‐1‐CD11b+ cells, the ratio of arginase 1+ (M2 macrophages) and iNOS+ cells (M1 macrophages) was analysed in this group of cells.

### 2.13 Statistical analysis

The data were analysed by Graphad prism software (v.8.3.0). *P* value was calculated by the Student's t test when only two groups were compared. For multiples groups, the *P* value was calculated by one‐way ANOVA with Tukey's test as post hoc test. *P* values < .05 indicated significant differences.

## 3 RESULTS

### 3.1 IL‐7 increases after myocardial I/R injury

**Figure 1:**
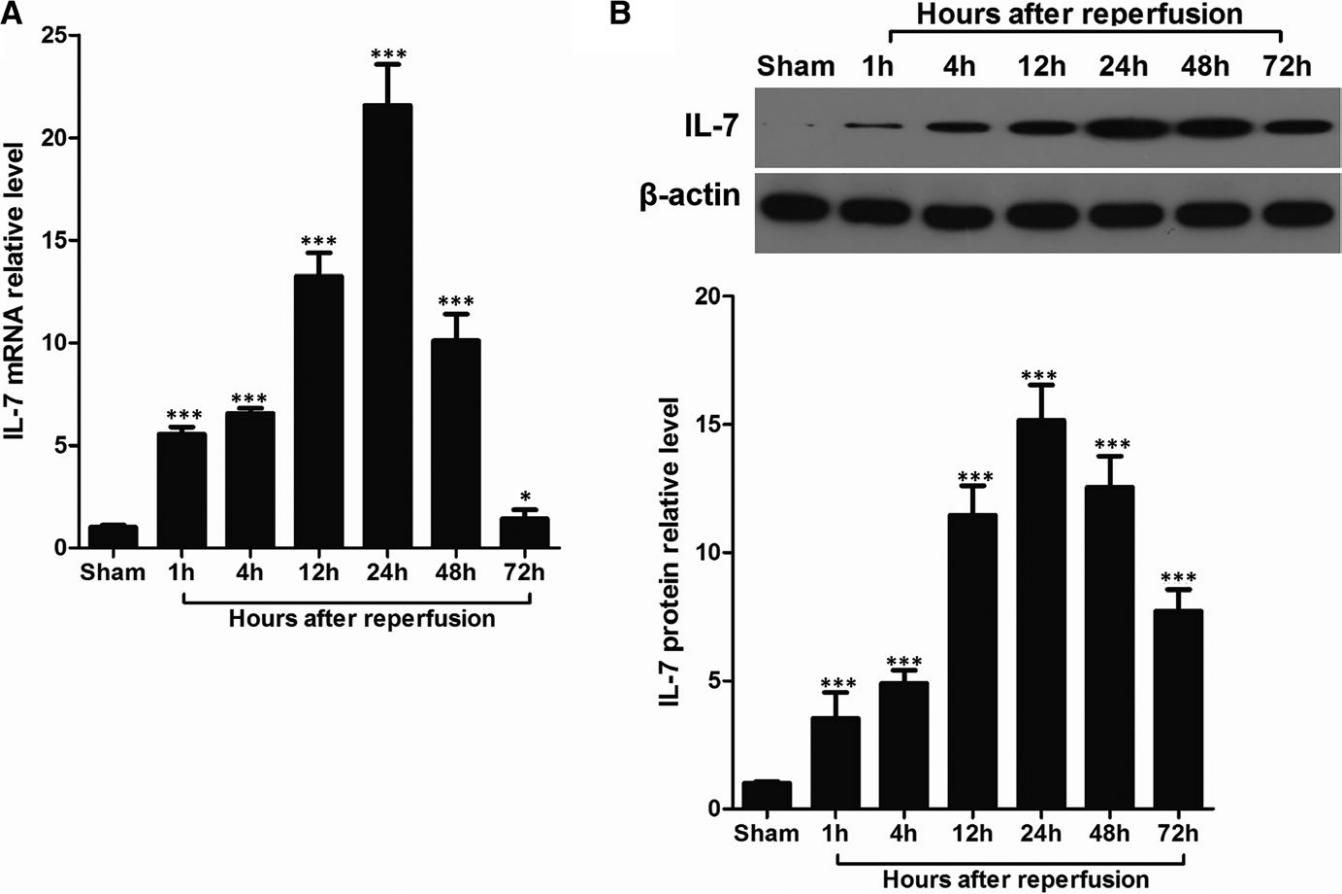
Induction of IL‐7 in heart tissues after I/R. A, Real‐time RT‐PCR analysis of the indicated IL‐7 mRNA levels in the heart tissues with 30 min of ischaemia and different time of reperfusion. B, Representative images of IL‐7 protein bands using Western blot in the heart tissues with 30 min of ischaemia and different time of reperfusion (upper), and statistical comparison of protein band grey values (lower). Data shown are mean ± SD (n = 5); Compared with sham group, * was *P* < .05 and *** was *P* < .001

The investigation of the IL‐7 involvement in myocardial I/R injury was initiated by the detection of dynamic changes in the IL‐7 mRNA expression using RT‐qPCR in mice heart tissues at 1, 4, 12, 24, 48 and 72 hours after reperfusion. As showed in Figure 1A, during the first 24 hours of reperfusion, IL‐7 mRNA underwent a rapid up‐regulation and peaked 24 hours after reperfusion. Then, IL‐7 mRNA level decreased rapidly after 24 hours of reperfusion and remained at a low level 72 hours after reperfusion. The dynamic changes in IL‐7 protein expression in heart tissues after reperfusion were similar. IL‐7 protein expression increased rapidly during the first 24 hours of reperfusion and then decreased (Figure 1B). However, unlike IL‐7 mRNA, IL‐7 protein level remained high 72 hours after reperfusion.

### 3.2 IL‐7 aggravates myocardial I/R injury

**Figure 2:**
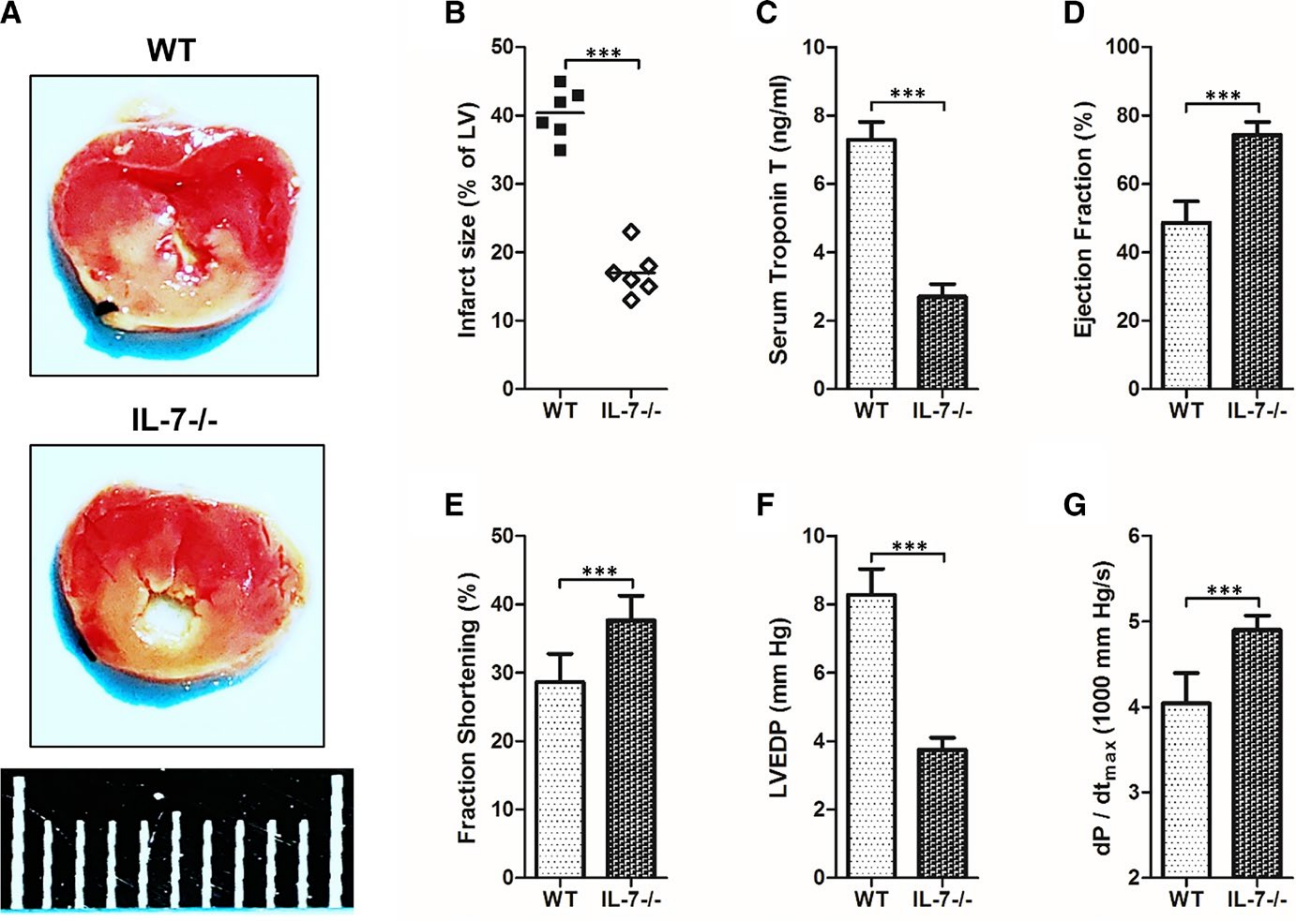
IL‐7 knockout attenuates myocardial I/R injury. The heart tissues of mice were harvested and being analysed at 24 h after reperfusion; (A, B) Representative images of left ventricular (LV) slices in normal mouse (WT) and IL‐7 knockout (IL‐7‐/‐) mouse at 24 hours after reperfusion (A), and the infract size of LV (white) is statistically compared (B); (C‐G) Serum troponin T (C), ejection fraction (D), fraction shortening (E), left ventricular end‐diastolic pressure (LVEDP) (F) and maximum systolic blood pressure (dP/dt_max_) (G) in WT and IL‐7‐/‐ mouse at 24 hours after reperfusion. Data shown are mean ± SD (n = 6). Comparison between the two groups, *** was *P* < .001. Scale bar = 1 cm

To study the effect of IL‐7 expression on the development of myocardial I/R injury, the myocardial I/R injury was compared between wild‐type (WT) C57BL/6 mice and IL‐7 knockout (IL‐7‐/‐) mice based on wild‐type C57 BL/6 mice by determining the infract size of left ventricular (LV) (Figure 2A,B), the serum troponin T level (Figure 2C), the ejection fraction (Figure 2D), the fraction shortening (Figure 2E), the left ventricular end‐diastolic pressure (LVEDP) (Figure 2F) and the maximum systolic blood pressure (dP/dt_max_) (Figure 2G). The results showed that the LV infract size, serum troponin T level and LVEDP in IL‐7‐/‐ mice were all significantly lower than in WT mice. In contrast, the ejection fraction, fraction shortening and dP/dt_max_ in IL‐7‐/‐ mice were all significantly higher than in WT mice.

**Figure 3:**
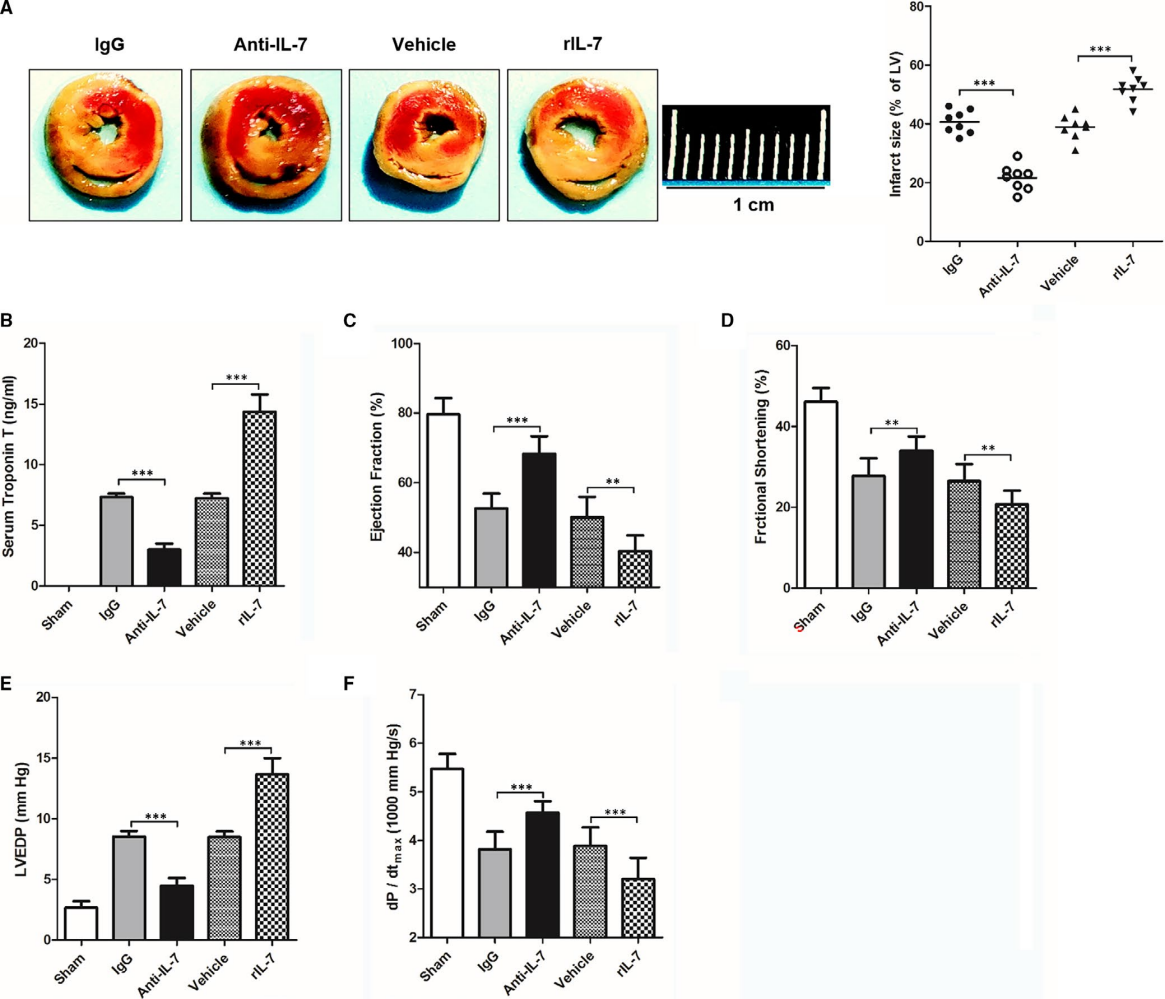
Effects of IL‐7 neutralization and supplementation on myocardial I/R injury in mice. The heart tissues of mice were harvested and being analysed, and cardiac function and haemodynamic measurements at 24 h after reperfusion; (A) Representative images of left ventricular (LV) slices in sham group, IL‐7 antibody isotype IgG treatment group (IgG), IL‐7 antibody treatment group (Anti‐IL‐7), solvent group (vehicle) and recombinant IL‐7 supplement group (rIL‐7) (left), and the infract size of LV (white) is statistically compared (right); (C‐G) Serum troponin T (B), ejection fraction (C), fraction shortening (D), left ventricular end‐diastolic pressure (LVEDP) (E) and maximum systolic blood pressure (dP/dt_max_) (F) in different group mouse at 24 hours after reperfusion. Data shown are mean ± SD (n = 8). Comparison between the two groups, *** was *P* < .001. Scale bar = 1 cm

To determine whether IL‐7 could be a potential target for the myocardial I/R injury treatment, myocardial I/R mice were treated systemically with neutralizing anti‐IL‐7 monoclonal antibody (mAb) or recombinant IL‐7 (rIL‐7) before reperfusion. Myocardial injury markers and cardiac function were measured 24 hours after reperfusion because the IL‐7 expression peaked in this time‐point after reperfusion. As shown in Figure 3A, treatment with neutralizing anti‐IL‐7 antibody led to a significantly decrease in the LV infract size ratio, whereas this ratio increased significantly after exogenous rIL‐7 repletion. Meanwhile, the levels of myocardial injury biomarkers and serum troponin T (Figure 3B) increased significantly after I/R injury. Neutralizing anti‐IL‐7 antibody treatment significantly reduced these levels, whereas exogenous rIL‐7 repletion increased them significantly. To study the IL‐7 effects on cardiac function following I/R injury, ejection fraction (Figure 3C), fraction shortening (Figure 3D), LVEDP (Figure 3E) and dP/dt_max_ (Figure 3F) were also evaluated. The data showed that the ejection fraction, fraction shortening and dP/dt_max_ were all significantly decreased after I/R injury, whereas LVEDP was significantly increased. Importantly, neutralizing anti‐IL‐7 antibody administration was able to reverse these changes, whereas exogenous rIL‐7 repletion significantly enhanced them.

### 3.3 IL‐7 promotes I/R‐induced cardiomyocyte apoptosis

**Figure 4:**
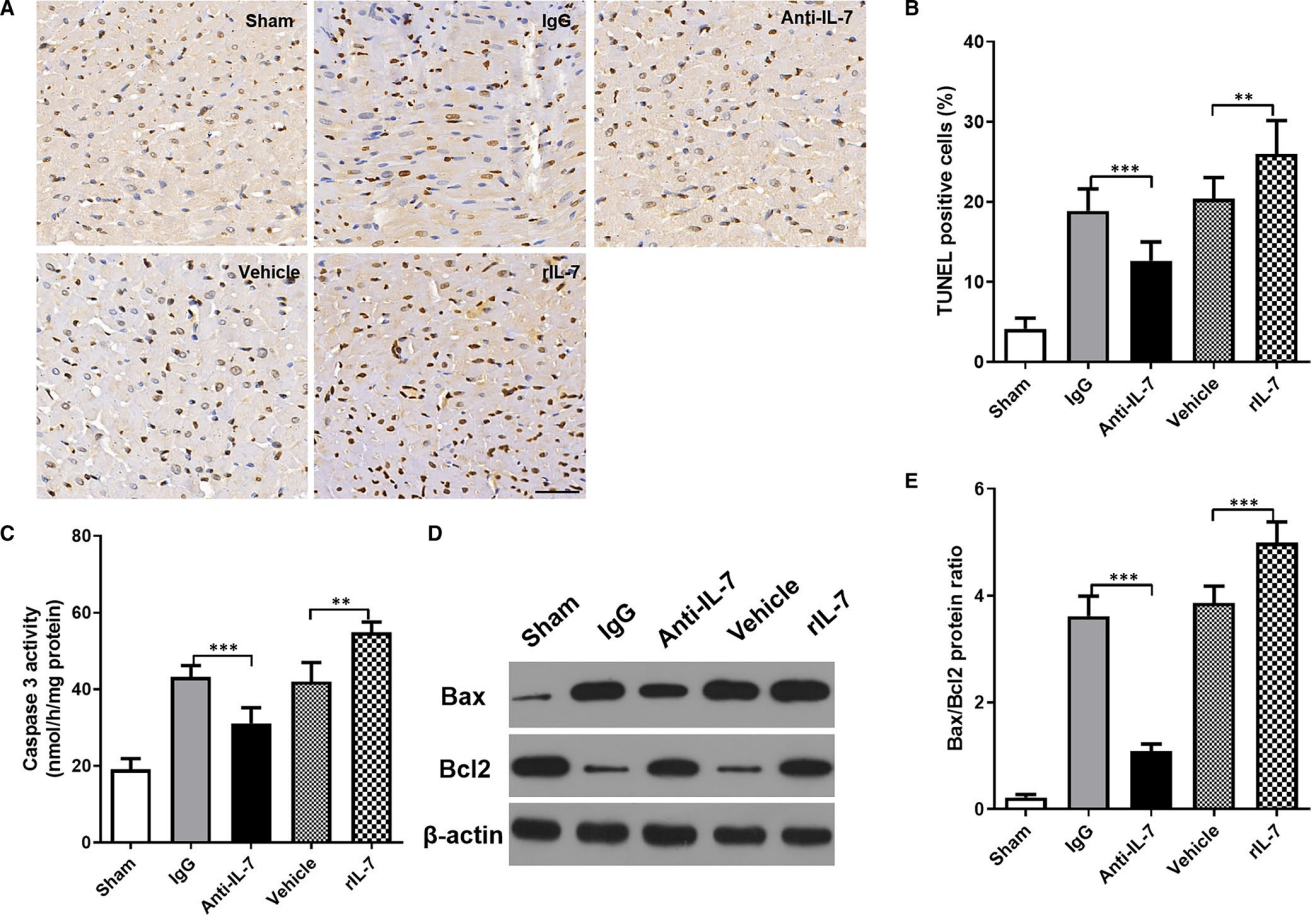
Effects of IL‐7 neutralization and supplementation on cardiomyocyte apoptosis in mice. (A, B) TUNEL staining was used to detect the apoptosis in the heart tissues at 24 hours after reperfusion (A), and statistical comparison of TUNEL positive cells (brown staining) (B); (C) The caspase‐3 activity of the heart was measured and compared; (D, E) Representative images of Bax and Bcl2 protein brands using Western blot in the heart tissues (D), statistical comparison of protein band grey values (E). Data shown are mean ± SD (n = 8). Comparison between the two groups, *** was *P* < .001

Apoptosis contributes greatly to I/R injury and is a direct factor that causes changes in cardiac function and myocardial injury.^24^ Therefore, to study the IL‐7 mechanism in the I/R injury, apoptosis in the LV section of heart tissues 24 hours following I/R injury was assessed using TdT‐mediated dUTP Nick‐End Labeling (TUNEL) staining. In this study, the apoptosis cells showed brown staining (Figure 4A). The apoptosis cell number was significantly increased following I/R injury in the LV section of heart tissues 24 hours after reperfusion. Neutralizing anti‐IL‐7 antibodies can significantly protect against apoptosis, whereas exogenous rIL‐7 repletion significantly promoted apoptosis after I/R injury. Results obtained with other apoptosis markers support the TUNEL staining results. Caspase 3 activity (Figure 4C) and Bax/Bcl2 protein expression ratio (Figure 4D,E) increased significantly following I/R injury in the LV section of heart tissues 24 hours after reperfusion. Similar to TUNEL staining results, neutralizing anti‐IL‐7 antibody treatment significantly reduced both markers, whereas the exogenous rIL‐7 repletion significantly enhanced them.

**Figure 5:**
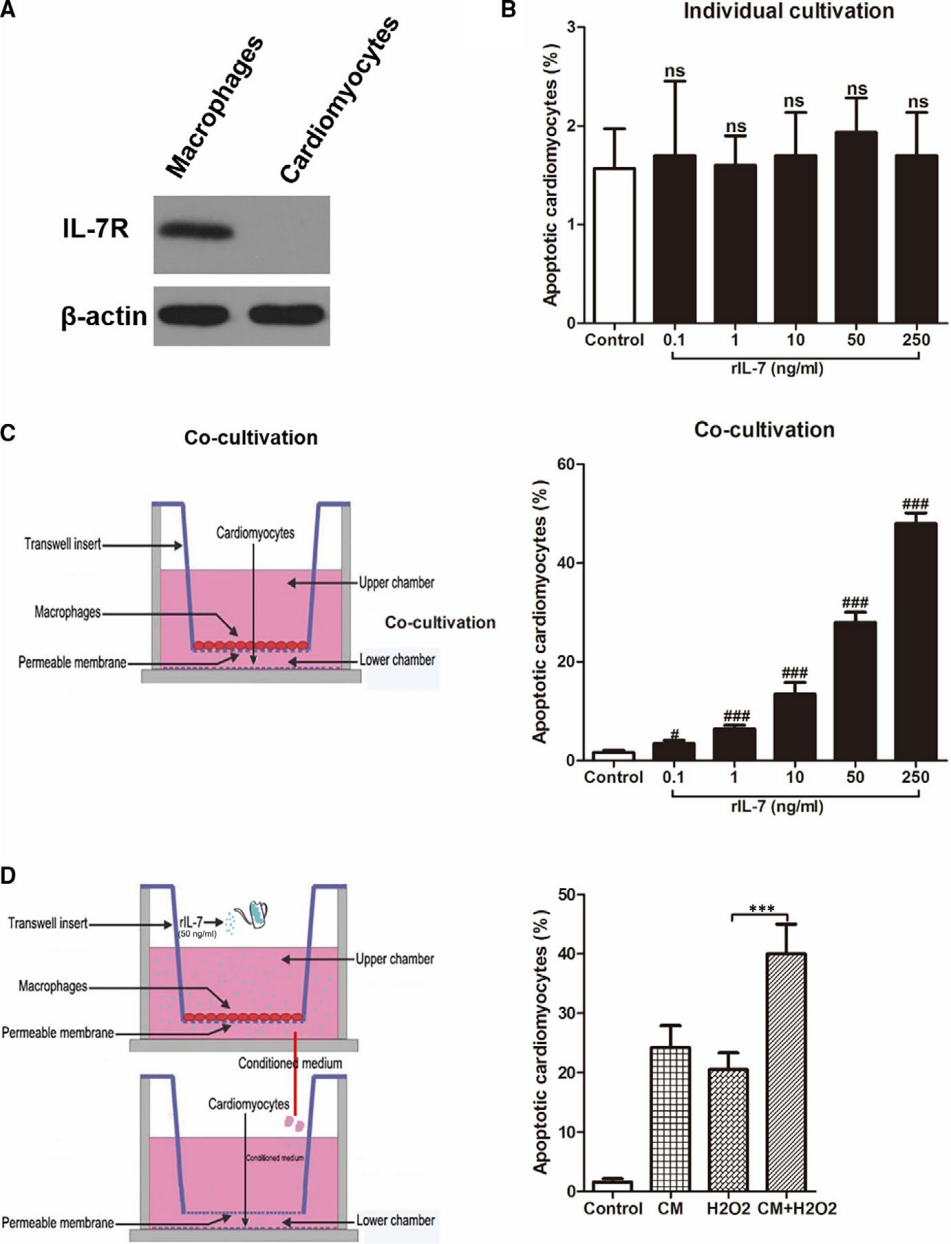
IL‐7‐induced cardiomyocyte apoptosis requires mediation of macrophages. (A) Western blot analysis of the indicated IL‐7 receptor (IL‐7R) protein levels in mouse peritoneal macrophages and mouse neonatal cardiomyocyte; (B, C) Mouse neonatal cardiomyocyte apoptosis under different doses of IL‐7 in a separate culture system for cardiomyocytes (B), or in a co‐culture system of macrophages and cardiomyocytes (C); (D) The conditioned medium was obtained by adding 50 ng/mL rIL‐7 to the macrophage culture medium for 24 h, and then using this conditioned medium to culture the mouse neonatal cardiomyocyte. 24 h after adding 100 μmol/L H_2_O_2_, mouse neonatal cardiomyocyte was harvested and analysed for apoptosis. Data shown are mean ± SD, and 3 independent replicates for each experiment. ns was *P* > .05, # was *P* < .05 and ### was *P* < .001 versus Control group. Comparison between the two groups, *** was *P* < .001

Previous studies have shown that IL‐7 helps immune cells to resist apoptosis, such as NK,^25^ T ^26^ and B cells.^27^ However, there are no previous reports on the IL‐7 expression effects on apoptosis in cardiomyocytes. It is well known that only when the IL‐7 receptor (IL‐7R) is expressed on the surface of a cell is that IL‐7 can affect the biological characteristics of this cell by binding to IL‐7R. Therefore, IL‐7R expression in mouse peritoneal macrophages and mouse neonatal cardiomyocytes was determined and revealed that IL‐7R was expressed on the surface of macrophages cells, but not on the cardiomyocyte surface (Figure 5A). Moreover, there were no significance differences in cardiomyocytes apoptosis between control group without any treatment and the groups treated with different concentrations of rIL‐7 (0, 0.1, 1, 10, 50 and 250 ng/mL) (Figure 5B). These data suggested that IL‐7 is not able to directly cause myocardial apoptosis. Inflammation caused by macrophage infiltration is one of the main factors that cause cardiomyocyte injury. Thus, it was suggested that IL‐7 would induce cardiomyocyte apoptosis via macrophage mediation. To test this hypothesis, macrophages and cardiomyocytes co‐culture systems were stablished and treated with different rIL‐7 concentrations (0, 0.1, 1, 10, 50 and 250 ng/mL). After 24 hours of incubation, cardiomyocyte apoptosis was evaluated. As shown in Figure 5C, rIL‐7 induced cardiomyocyte apoptosis in a dose‐dependent manner. Furthermore, a macrophage conditioned medium (CM) was prepared by adding 50 ng/mL rIL‐7 into macrophages medium. CM was used to culture cardiomyocytes and was able to further increase their apoptosis (Figure 5D).

### 3.4 IL‐7 increases macrophage infiltration into heart tissues following I/R

**Figure 6:**
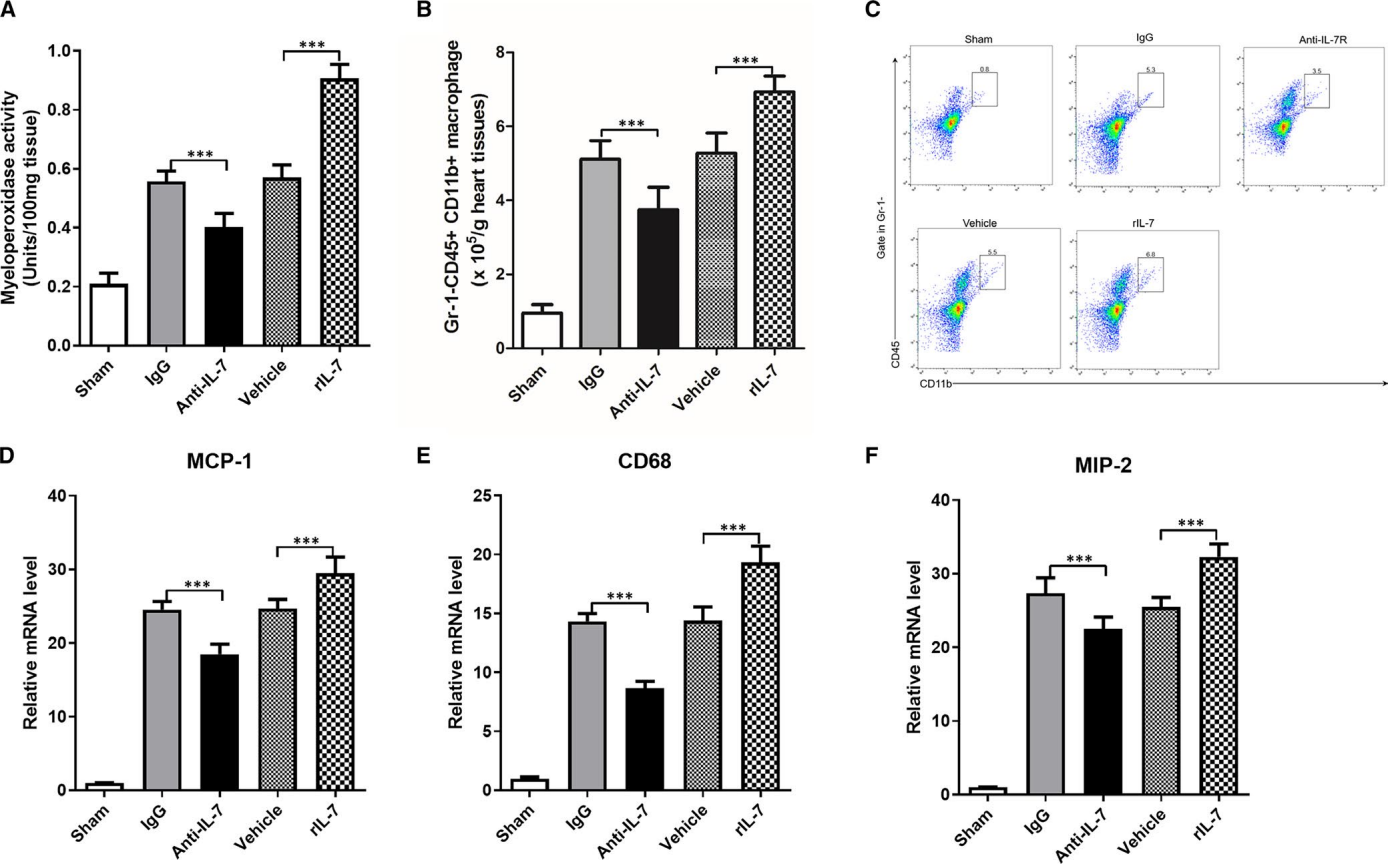
IL‐7 increases macrophage infiltration in heart tissue following I/R. (A) Cardiac myeloperoxidase activity in heart tissues at 24 hours after reperfusion; (B‐C) Statistical comparison of CD45 + Gr‐1‐CD11b+ macrophage in heart tissues at 24 hours after reperfusion (B), and representative flow analysis images (C); (D‐F) Real‐time RT‐PCR analysis of the indicated CD68 (D), monocyte chemotactic protein 1 (MCP‐1) (E) and macrophage inflammatory protein‐2 (MIP‐2) (F) mRNA levels in heart tissues at 24 h after reperfusion. Data shown are mean ± SD (n = 8). Comparison between the two groups, *** was *P* < .001

Macrophage infiltration is a hallmark pathological change in early stage of I/R injury and one of the main causes of myocardial damage. As determined by MPO activity and fluorescence activated cell sorting analysis of CD45 + Gr‐1‐CD11b + macrophages, I/R injury induced MPO activity and significantly increased the CD45 + Gr‐1‐CD11b + macrophage number. Neutralizing anti‐IL‐7 antibodies were able to significantly decrease MPO activity and the CD45 + Gr‐1‐CD11b + macrophage number in the heart tissues 24 hours after reperfusion, whereas exogenous rIL‐7 repletion increased them significantly (Figure 6A‐C). Monocyte chemotactic protein 1 (MCP‐1) is a small cytokine belonging to the CC chemokine family that can recruit monocyte macrophages to the injured tissue.^28^ CD68 is a specific marker for macrophages, and macrophage inflammatory protein‐2 (MIP‐2) is secreted mainly by macrophages, nerve cells and endothelial cells.^29^ Therefore, the evaluation of MCP‐1, CD68 and MIP‐2 expression can characterize macrophage infiltration. As expected, the I/R injury induced a high expression of MCP‐1, CD68 and MIP‐2 mRNA in the heart tissues. Neutralizing anti‐IL‐7 antibody administration was able to significantly decrease MCP‐1, CD68 and MIP‐2 mRNA levels in heart tissues 24 hours after reperfusion, whereas exogenous rIL‐7 repletion increased them significantly (Figure 6D‐F).

**Figure 7:**
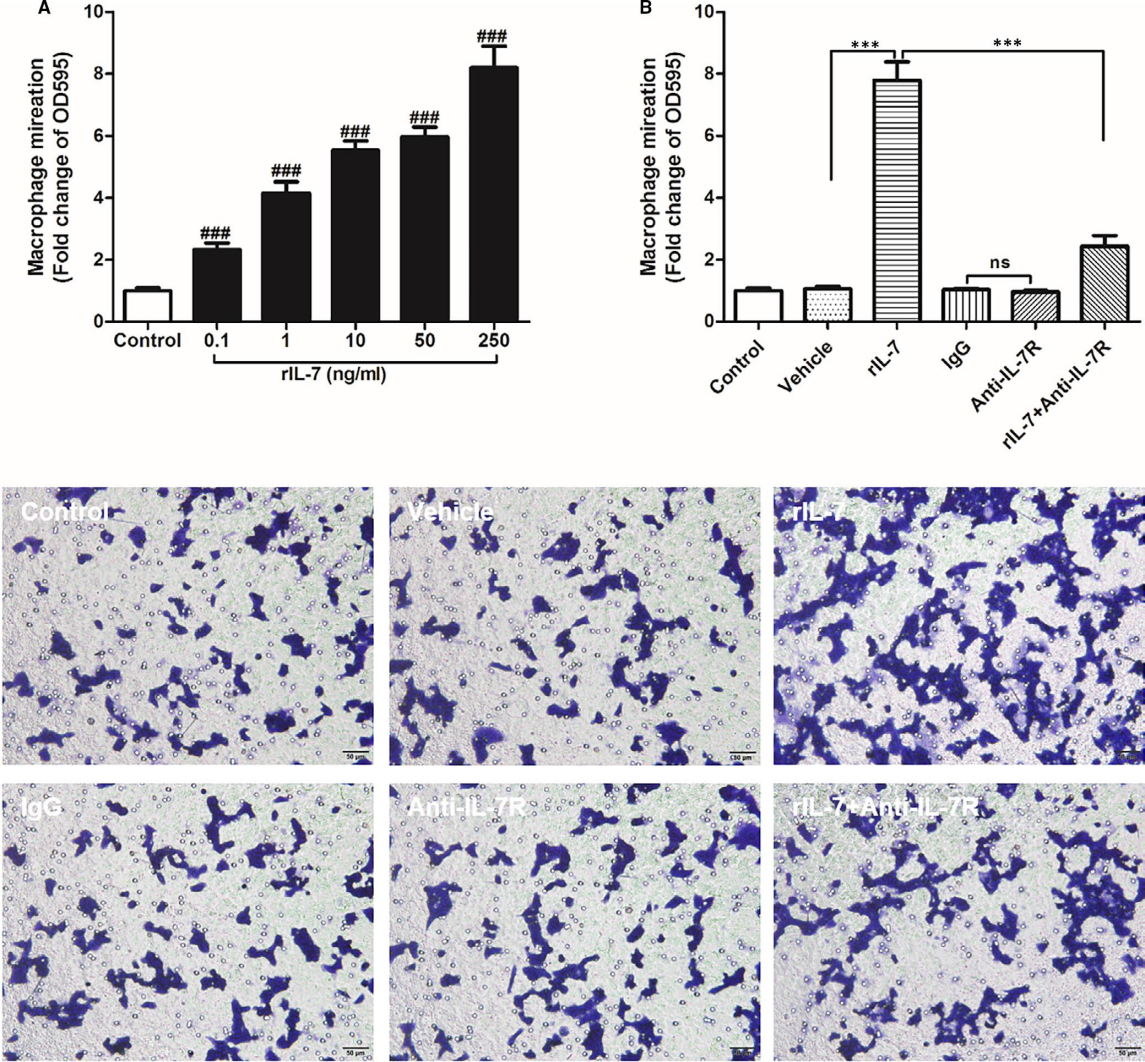
IL‐7 promotes macrophage migration in vitro. A, Mouse peritoneal macrophages migration under different doses of IL‐7 in a separate culture system, and data were expressed as fold change of OD595 versus control group. B, Effect of different treatment on the migration of mouse peritoneal macrophages. Control, without any treatment; Vehicle, by adding solvents that dissolve rIL‐7; rIL‐7, by adding 50 ng/mL rIL‐7; IgG, by adding IL‐7R antibody isotype IgG; Anti‐IL‐7R, by adding 10 mg/mL anti‐IL‐7R antibody; rIL‐7 + Anti‐IL‐7R, first added 10 mg/mL anti‐IL‐7R antibody for 2 hours and then added 50 ng/mL rIL‐7 for 24 hours. ### was *P* < .001 versus control group. Comparison between the two groups, *** was *P* < .001. Data shown are mean ± SD, and three independent replicates for each experiment

The rIL‐7 effect on the ability of macrophages to migrate was evaluated in vitro and revealed that rIL‐7 promoted macrophage migration in a dose‐dependent manner (Figure 7A). Then, the rIL‐7 mechanism of promoting macrophage migration was analysed by neutralizing IL‐7R antibody treatment. The data showed that, although the use of IL‐7R neutralizing antibodies alone did not affect macrophage migration, IL‐7R neutralizing antibodies were able to significantly reduce the rIL‐7‐induced high macrophage migration (Figure 7B). Therefore, these data suggested that IL‐7 promoted macrophage migration via targeting IL‐7R.

### 3.5 IL‐7 alters T helper (Th)1 and Th 2 cytokines following I/R injury in vivo

**Figure 8:**
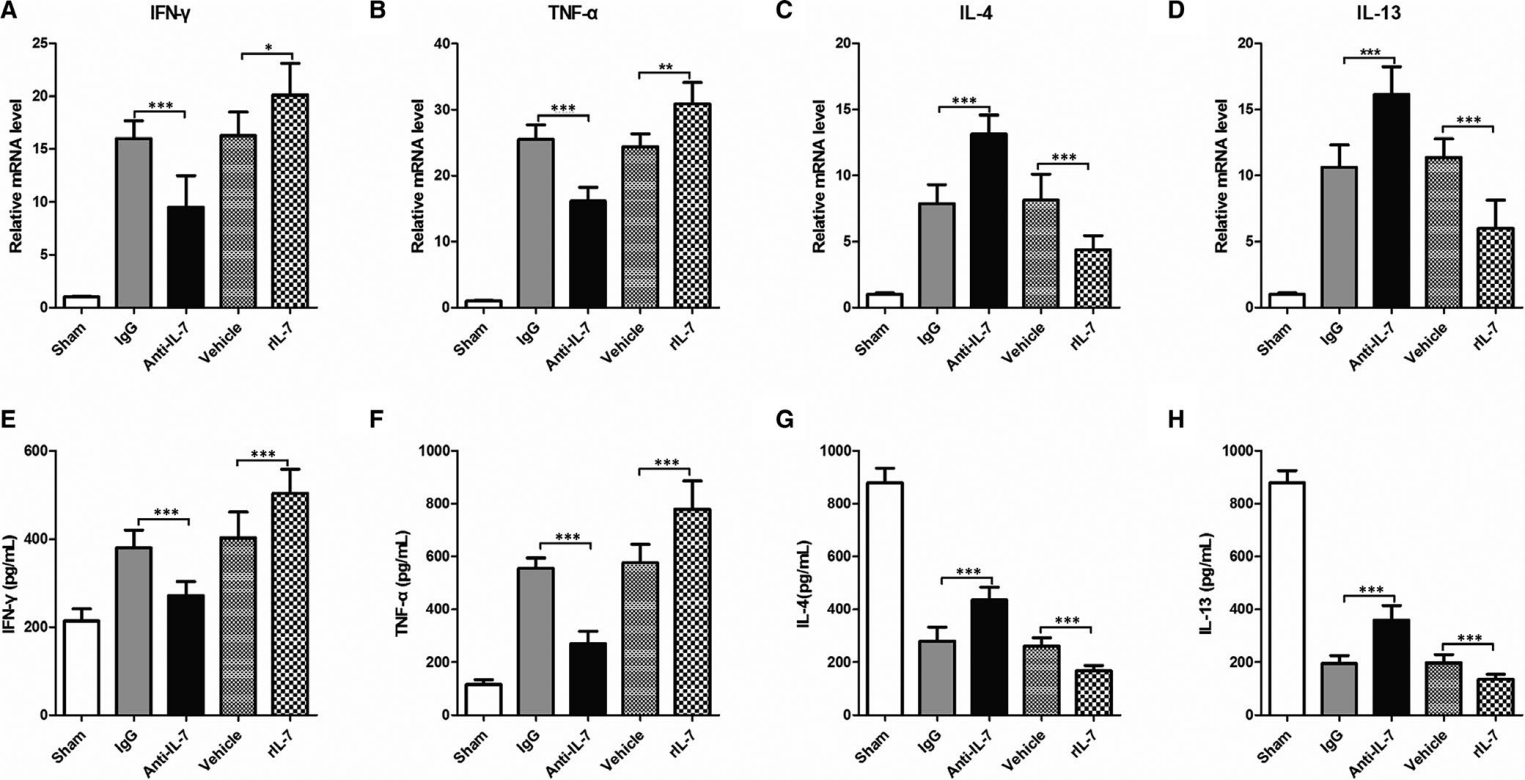
IL‐7 alters Th1/Th2 cytokines in heart tissue following by I/R. (A‐D), Real‐time RT‐PCR analysis of the indicated IFN‐γ (A), TNF‐α(B), IL‐4 (C) and IL‐13 (D) mRNA levels in the heart tissues; (E‐H) Enzyme‐linked immunosorbent assay was used to detect the protein levels of IFN‐γ (E), TNF‐α(F), IL‐4 (G) and IL‐13 (H) levels in the heart tissues. Data shown are mean ± SD (n = 8). Comparison between the two groups, * was *P* < .05, ** was *P* < .01 and *** was *P* < .001

To study the IL‐7 expression effects on the acute inflammatory response after I/R injury, the mRNA levels of IFN‐γ and TNF‐α (Th1 cytokines) and IL‐4 and IL‐13 (Th2 cytokines) were determined using RT‐qPCR. In addition, protein levels of IFN‐γ, TNF‐α, IL‐4 and IL‐13 were assessed by ELISA (Figure 8). The use of IL‐7 neutralizing antibody led to a reduction in the IFN‐γ and TNF‐α mRNA levels, and an increase in the IL‐4 and IL‐13 levels in heart tissues following I/R injury 24 hours after reperfusion (Figure 7A‐D). In contrast, exogenous rIL‐7 repletion markedly increased the IFN‐γ and TNF‐α mRNA levels and decreased the IL‐4 and IL‐13 levels. Remarkably, the results observed in the mRNA level evaluation were also observed in the assessment of IFN‐γ, TNF‐α, IL‐4 and IL‐13 protein levels (Figure 7E‐H).

### 3.6 IL‐7 promotes M2 macrophage activation in heart tissues following I/R injury in vivo

**Figure 9:**
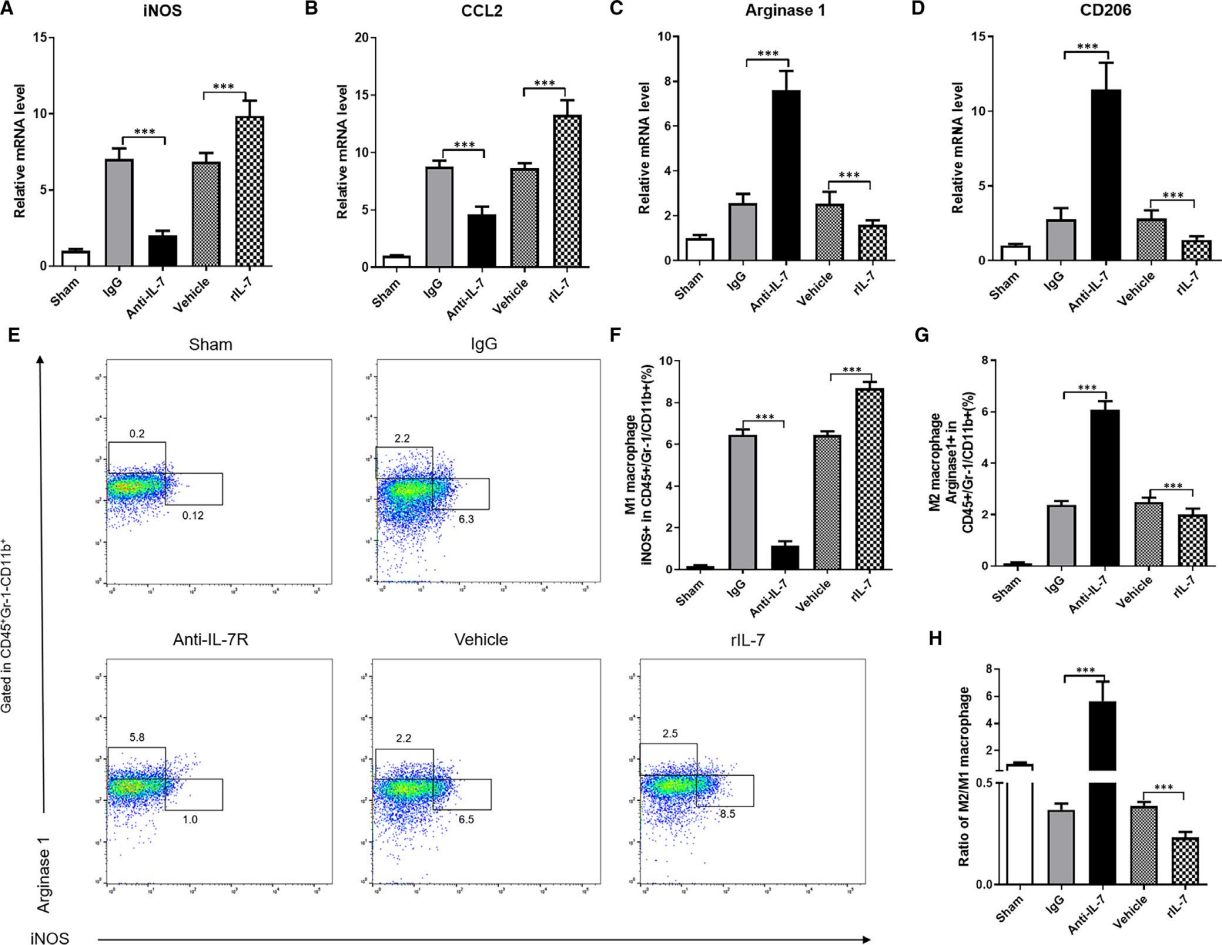
IL‐7 promotes M2 macrophage activation in the heart tissues in heart tissue following by I/R. (A‐D), Real‐time RT‐PCR analysis of the indicated iNOS (A), CCL2 (B), Arginase 1(C) and CD206 (D) mRNA levels in the heart tissues; (E‐H) Representative flow cytometry data of activated macrophages in the heart tissues of different group (E), statistical analysis of the total number of activated M1 macrophages (F), M2 macrophages (G) and the ratio of M2/M1macrophages (H). Data shown are mean ± SD (n = 8). Comparison between the two groups, *** was *P* < .001

Macrophages have two phenotypes, namely M1 macrophages (pro‐inflammatory) and M2 macrophages (anti‐inflammatory). Under physiological conditions, M1 and M2 macrophages maintain a physiological balance, which is broken during tissue damage. To determine the IL‐7 effects on the physiological balance of macrophage phenotypes in the heart tissues following I/R injury, the mRNA levels of iNOS and CCL2 (specific marker of M1 macrophages), and arginase 1 and CD206 (specific marker of M2 macrophages) were measured. The use of anti‐IL‐7 antibody significantly decreased the iNOS and CCL2 mRNA levels, whereas significantly increased the arginase 1 and CD206 levels in heart tissues following I/R injury 24 hours after reperfusion (Figure 9A‐D). On the other hand, exogenous rIL‐7 repletion markedly increased the iNOS and CCL2 mRNA levels, whereas significantly reduced the arginase 1 and CD206 levels. Flow cytometry analysis revealed that the use of anti‐IL‐7 can significantly reduce the iNOS positive macrophages number in CD45 + Gr‐1‐CD11b+ cells (Figure 9E‐F) and markedly increase arginase 1 positive macrophage number in CD45 + Gr‐1‐CD11b+ cells (Figure 9E‐G). Remarkably, the endogenous IL‐7 neutralization and the exogenous IL‐7 repletion altered the physiological balance of M1 and M2 macrophages in heart tissues after I/R injury. That is, the endogenous IL‐7 neutralization increased the ratio of M2/M1 macrophages, whereas the exogenous IL‐7 repletion decreased it (Figure 9H). This evidence show that IL‐7 can promote macrophage differentiation into M1 macrophages and decrease the M2/M1 macrophage ratio in heart tissues after I/R injury.

## 4 DISCUSSION

In the present study, we revealed the important role of IL‐7 in mediating myocardial I/R injury in mice. The I/R injury induced a rapid increase of IL‐7 expression in heart tissue. The IL‐7 knockout or endogenous IL‐7 neutralization alleviated I/R injury in vivo. In addition, anti‐IL‐7 treatment in vivo reduced cardiomyocyte apoptosis, macrophage infiltration and promoted M2 macrophage polarization in mice heart tissue following I/R injury. On the other hand, exogenous IL‐7 repletion induced opposite effects. In vitro, IL‐7 was not able to cause cardiomyocyte apoptosis directly, but through macrophages, in addition to promote their migration.

Inflammation plays a pivotal role in the development and progression of I/R injury. Myocardial I/R injury induces the production and secretion of inflammatory cytokines, chemokines and adhesion factors that aggravating myocardial I/R injury. In contrast, the inhibition of excessive inflammatory responses protects myocardial cells from apoptosis and necrosis, maintains the normal physiology of endothelial cells and reduces myocardial infarction area.^14^ Notably, immune cells play an important role in the I/R injury‐induced inflammation. During ischaemia‐reperfusion, neutrophils bind to adhesion factors in vascular endothelial cells and cardiomyocytes, release many chemotaxis inflammatory mediators and can amplify the inflammation cascade to cause vascular dysfunction and tissue damage.^30^, ^31^ Macrophages, especially M1 macrophages, have increased infiltration into tissues, which is an important pathological manifestation of tissue inflammation and a major cause of tissue damage.^32^, ^33^ Emerging evidence from experimental and clinical studies indicates that IL‐7 acts as a mediator between immune cells and inflammation through regulation of chemotaxis, proliferation, survival and differentiation of immune cells.^34^, ^35^ These findings have led to a new understanding of the IL‐7 role in inflammation‐related diseases, such as chronic colitis,^36^ rheumatoid arthritis ^37^ and infectious diseases. Importantly, IL‐7 was detected in intestinal I/R in vitro and in vivo ^38^ and was identified as an expansion and activation driver of CD4 + CD28null T cells in myocardial infarction patients.^39^, ^40^ Here, we found that IL‐7 expression increased rapidly in the early post‐reperfusion period and peaked in 24 hours, which suggests that IL‐7 may be involved in the myocardial I/R injury in the early period.

To investigate the IL‐7 mechanism in the myocardial I/R injury, we measured apoptosis in heart tissues after I/R injury. Although there are many mechanisms that cause I/R injury, such as inflammation, oxidative stress and mitochondrial damage, cardiomyocyte apoptosis is a direct I/R injury physiological manifestation.^41^, ^42^ The data from the present study showed that endogenous IL‐7 neutralization reduced apoptosis and apoptosis‐related marker levels in heart tissues following I/R injury. In contrast, exogenous IL‐7 repletion induced opposite effects. This indicates that IL‐7 aggravated myocardial I/R injury by promoting cardiomyocyte apoptosis. It is well known that IL‐7 must bind to its receptor (IL‐7R) to induce biological effects. However, we found no IL‐7R expression in neonatal mouse cardiomyocytes. Most importantly, rIL‐7 was not able to directly induce cardiomyocyte apoptosis. Nevertheless, rIL‐7 induced cardiomyocyte apoptosis in macrophage/cardiomyocyte co‐culture system and macrophage‐conditioned medium was able to promote H_2_O_2_‐induced cardiomyocyte apoptosis. Therefore, these results indicate that cardiomyocyte apoptosis worsened by IL‐7 following I/R injury is at least partially macrophage‐dependent.

Ischaemia‐reperfusion exacerbates tissue injury by recruiting immune cells to the damaged tissues, including neutrophils, macrophages and T cells, through the generation and release of various cytokines, chemokines and adhesion molecules.^43^ Neutrophils infiltrate the ischaemic area a few hours after ischaemia‐reperfusion and reach their peak on the first day. Subsequently, a large number of monocytes infiltrate the ischaemic area and this infiltration lasts for two weeks. Lymphocytic infiltration, in turn, appears in the late ischaemia‐reperfusion phase, so that monocytes and their differentiated macrophages are the inflammatory cells that cause I/R damage and regulate tissue.^44^ Meanwhile, macrophages are the inflammatory cells with the longest residence time in repairing myocardial tissue damage after I/R injury.^2^, ^3^ The macrophage infiltration triggers the release of a series of chemokines and increases the expression of adhesion molecules in the intrinsic cells of the heart. This results in the amplification of the inflammatory cascade in the heart, thereby exacerbating myocardial I/R injury.^12^, ^13^ In this study, we found that IL‐7 neutralization can significantly reduce the macrophage infiltration in cardiac tissues following I/R injury in vivo, as well as we found that rIL‐7 promoted macrophage migration in vitro. Therefore, IL‐7 exacerbates myocardial I/R injury by activating macrophages and promoting macrophage migration to damaged tissues.

Macrophages have two phenotypes, namely M1 and M2. M1 macrophages have iNOS as their specific marker and can promote inflammation by secreting pro‐inflammatory factors (IL‐1β, IL‐6, IL‐12, TNF‐α) and chemokines (MCP‐1). In contrast, M2 macrophages have arginase 1 as their specific marker and exert anti‐inflammatory effects by producing large amounts of the anti‐inflammatory factor IL‐10 and inhibiting the secretion of pro‐inflammatory factors IL‐12, IL‐1 and TNF‐α.^45^, ^46^ Macrophage differentiation and activation depends on specific growth and differentiation factors, receptors, signalling pathways and transcription factors.^47^, ^48^ In a normal physiological environment, M1 and M2 macrophages in living organs are in balance, whereas this balance is broken in damaged tissues and organs, such as in heart tissue following by I/R.^30^, ^31^ In the process of ischaemia‐reperfusion injury, neutrophils bind with adhesion factors on vascular endothelial cells and cardiomyocytes to release many inflammatory/anti‐inflammatory mediators with chemotactic effects.^12^ Therefore, in this study, pro‐inflammatory mediators play a major role in the heart tissue after I/R injury without intervention, so macrophages are polarized to M1 macrophages. In addition, cytokines secreted by T helper cells (Th) have macrophage polarization as their main role. According to the difference in secreted cytokines, Th cells are divided into two types, Th1 and Th2. Th1 cells are characterized by secreting cytokines, such as interferon Y, IL‐1, IL‐2 and TNF‐a, which polarizes macrophages into M1 macrophages. Th2 cells secrete IL‐4, IL‐5, IL‐6, IL‐10 and IL‐13, which polarizes macrophages into M2 macrophages.^47^, ^48^ Based on these data, we investigated the expression of some markers related to macrophage polarization and phenotype. We found that the endogenous IL‐7 neutralization increased the ratio of M2/M1 macrophages, whereas the exogenous IL‐7 repletion decreased it in heart tissues following I/R injury. The data showed that IL‐7 promoted polarization of M2 macrophages.

In conclusion, the data from this study suggest that IL‐7 is produced rapidly in heart tissues during early ischaemia‐reperfusion and aggravates myocardial I/R injury by regulating macrophage infiltration and polarization. Our study also shows that the control of endogenous IL‐7 levels immediately after ischaemia‐reperfusion can help to reduce myocardial I/R injury.

## ACKNOWLEDGEMENT

We are appreciative to Wei Gong for his help in the research.

## CONFLICT OF INTEREST

None.

## AUTHOR CONTRIBUTIONS

**Mengwen Yan:** Data curation (equal); Formal analysis (equal); Investigation (equal); Resources (equal); Software (equal). **Yaliu Yang:** Formal analysis (equal). **Ying Zhou:** Investigation (equal); Methodology (equal). **Changan Yu:** Validation (equal). **Rui Li:** Investigation (equal); Resources (equal); Software (equal). **Wei Gong:** Conceptualization (equal); Funding acquisition (equal); Project administration (equal); Writing‐review & editing (equal). **Jingang Zheng:** Conceptualization (equal); Funding acquisition (equal); Project administration (equal); Writing‐original draft (equal); Writing‐review & editing (equal).

## Data Availability

The data that support the findings of this study are available from the corresponding author upon reasonable request.
